# Assessing Future Climate Change Impacts on Potato Yields — A Case Study for Prince Edward Island, Canada

**DOI:** 10.3390/foods12061176

**Published:** 2023-03-10

**Authors:** Toyin Adekanmbi, Xiuquan Wang, Sana Basheer, Rana Ali Nawaz, Tianze Pang, Yulin Hu, Suqi Liu

**Affiliations:** 1 Canadian Centre for Climate Change and Adaptation, University of Prince Edward Island, St. Peters Bay, Charlottetown, PE C0A 2A0, Canada; toadekanmbi@upei.ca (T.A.); sbasheer10090@upei.ca (S.B.); rnawaz@upei.ca (R.A.N.); tpang11616@upei.ca (T.P.); sxliu@gov.pe.ca (S.L.); 2 School of Climate Change and Adaptation, University of Prince Edward Island, Charlottetown, PE C1A 4P3, Canada; 3 Faculty of Sustainable Design Engineering, University of Prince Edward Island, Charlottetown, PE C1A 4P3, Canada; yulinhu@upei.ca; 4 Department of Agriculture and Land, Government of Prince Edward Island, Charlottetown, PE C1A 7N8, Canada

**Keywords:** potato (*Solanum tuberusom*), climate change, drought, food security, heat, phenology, tuber

## Abstract

Crop yields are adversely affected by climate change; therefore, it is crucial to develop climate adaptation strategies to mitigate the impacts of increasing climate variability on the agriculture system to ensure food security. As one of the largest potato-producing provinces in Canada, Prince Edward Island (PEI) has recently experienced significant instability in potato production. PEI’s local farmers and stakeholders are extremely concerned about the prospects for the future of potato farming industries in the context of climate change. This study aims to use the Decision Support System for Agrotechnology Transfer (DSSAT) potato model to simulate future potato yields under the Coupled Model Intercomparison Project Phase 6 (CMIP6) climate scenarios (including SSP1–1.9, SSP1–2.6, SSP2–4.5, SSP3–7.0, and SSP5–8.5). The study evaluates the combined effects of changing climatic conditions at local scales (i.e., warming temperature and changing precipitation patterns) and increasing carbon dioxide (CO_2_) concentration in the atmosphere. The results indicate future significant declines in potato yield in PEI under the current farming practices. In particular, under the high-emission scenarios (e.g., SSP3–7.0 and SSP5–8.5), the potato yield in PEI would decline by 48% and 60% in the 2070s and by 63% and 80% by 2090s; even under the low-emission scenarios (i.e., SSP1–1.9 and SSP1–2.6), the potato yield in PEI would still decline by 6–10%. This implies that it is important to develop effective climate adaptation measures (e.g., adjusting farming practices and introducing supplemental irrigation plans) to ensure the long-term sustainability of potato production in PEI.

## 1. Introduction

Potato is the most common non-grain crop, belonging to the family *Solanaceae* [1,2]. Globally, potato is the fourth most recognized tuber food crop consumed, after maize, rice, and wheat, and is vital for food security because of its excellent potential for high yields and nutritional value [2,3]. Globally, potato is produced in over 155 countries, contributing nutritional value for more than a billion people [4,5]. Even though the global production is one-half that of wheat, rice, and maize, its production has increased by one-fifth since 1990, while its consumption has increased by more than double in developing countries [5,6]. In 2021, the Food and Agriculture Organization (FAO) estimated global potato yield to be 20.7 tonnes/hectare (t/ha), cultivated on about 18.2 million hectares (Mha) of land. The increase in potato production has significantly overtaken many other crops accounting for more than 50% of potato production globally [7]. Specifically, in 2021, Canada was ranked as one of the top twelve countries producing the highest potato yields of about 41.3 t/ha harvested from over 150,000 ha of land [8,9,10,11] and was ranked as the fifth and sixth largest exporter of fresh and seed potatoes, respectively, with 25% of the yields from Prince Edward Island (PEI) [9,12,13]. Potato is a major agricultural crop after canola, corn, wheat, and soybean, and a high-producing vegetable crop cultivated in Canada. In PEI, potato contributes majorly to the economy. It is cultivated on an average land size of 35,378 hectares (ha), producing about 36.8 t/ha yields annually [9]. Potato grows well in PEI because of its particular soil characteristics, i.e., red and rich in iron, and retains the required water during growth and development. Potato is grown during spring–summer (May to October) under rainfed conditions. Adequate rainfall with cold winters and warm summers with proper light, heat, and water balance contribute to the optimal quality potato yields in PEI. PEI is a major producer of potatoes exported from Canada [14].

Potato is a staple food consumed daily around the world and is categorized as a dietary vegetable containing many minerals and vitamins [15,16,17]. Potato contributes to the four pillars of food security, “access, availability, stability, and utilization,” for Sustainable Developmental Goal 2 (SDG 2-Zero Hunger). Approximately three-quarters of their total dry weight is in the form of starch, with amounts that depend on the variety [16]. Potatoes have a small protein content, but essential amino acids such as lysine and metabolites increase their biological value and utilization [16]. They are good sources of vitamins such as B6 and C and trace amounts of folate, thiamin, niacin, and riboflavin; 0.5–2% of dietary fiber is contained in potatoes with other minerals, such as magnesium, iron, potassium, and phosphorus [15,16]. Potatoes ensure food security and provide income and employment [18]. Potatoes from Canada, especially from PEI, are globally recognized and exported because of their safety and quality [19]. Assessing the impact of climate change (SDG 13-Climate Action) on potato yields is crucial to enhancing food security.

Potato is one of the most vulnerable crops in changing climates, with events such as long-lasting droughts, extreme heat, and unanticipated frosts [5,20]. The temperature is expected to increase as the climate changes, with inconsistent precipitation patterns. Climate change is impacting the frequency and intensity of extreme climate events, and SDG 2 (Zero Hunger) can be achieved by addressing SDG 13 (Climate Action) [13,21,22]. Although crop management practices cause about 67% of the variations in potato yields, climate change is a significant challenge faced by the agricultural sector [13]. Potato yields likewise depend on factors such as water and soil management practices, seed quality, chemical and bio-fertilization, soil moisture contents, elevation, slope, and supplement irrigation [13]. Potato development stages, such as sprouting, emergence, and leaf area development, are temperature sensitive. Temperature thresholds and photoperiod sensitivity are vital in determining the development of potatoes and initiating potato tuber induction vary with potato varieties [23]. Potato is a temperate crop that thrives between 16 °C and 19 °C if 20 to 24 inches of water requirement are fulfilled; however, when the temperature exceeds 30 °C, it can cause slow tuber initiation and development and physical damage to the tubers [24,25,26,27,28]. Surface temperatures below 0 °C during potato development causes frost, which burns stems, leaves, and potato cell organelles to form soft and blackened parts [29,30]. Considering the potato phenology, climate change can cause an advancement or delays in the emergence, tuber initiation, bulking, and maturity of potatoes, determined by regional location. Likewise, the emergence and drops of leaves could be early or delayed [31,32]. Potato requires 400 to 800 mm of rain/water, which invariably depends on meteorological variables and other factors [26,33]. Water shortage beyond 60% to 65% causes drought that reduces the growth rate, while excessive water causes leaching and tissue decay inside potato tuber called blackheart [26,34]. Considering previous literature, carbon dioxide (CO_2_) is reported to be beneficial to potatoes, causing increased photosynthesis rates that make potatoes bulk faster [35,36,37].

Meanwhile, the elevation of CO_2_ increases the rate of potato susceptibility to pests and diseases and yields phenology, causing interferences between implemented and natural biological processes [20,38]. In addition to abiotic factors, biotic factors such as pests, nematodes, and pathogens can affect potato yields [26,39]. Water stress conditions can likewise affect optimal potato yields [26,38,40]. Due to the effect of the changing climate conditions on crops in PEI, there is a dire need to assess the impacts of climate variables, such as temperature, precipitation, and CO_2_, on potato yields [41,42,43]. The development of adaptation strategies relies on understanding the effect of farming practices, genetics, and thermal trends on potato cultivation [5,20,44,45]. Hence, the need to assess the climate change impacts to strategize a coping mechanism for the cultivation of potatoes in PEI.

Recently, there has been high interest in using various crop–weather models to estimate the impacts of climatic changes on potatoes. Crop simulation models (CSMs) are an essential tool that uses input datasets with future emission scenarios to evaluate the potential effects of climate change on crops, e.g., the Decision Support System for Agrotechnology Transfer (DSSAT) model. Many studies use the DSSAT model to assess the effects of climate change on potatoes [31,44,46]. Only a few studies use the model to assess variations in the Canadian province’s potato yields [47]. In addition, Coupled Model Intercomparison Project Phase 6 (CMIP6) data have been used to assess the impacts of climate change on crops [48,49]. Many other studies use different methods and tools to assess potato yield response to climatic variables; for instance, Maqsood et al. (2020) [13] used ClimPACT2, and Jiang et al. 2021 [50] used analysis of variance (ANOVA) and second-order polynomial regressions to assess potato yield response to climate change and water. Overall, a study has yet to be conducted in PEI to assess climate change impacts on potato yields using physically based crop models such as the DSSAT model. In addition, the climate change scenarios used in previous studies are from the CMIP3 or CMIP5 datasets rather than the latest CMIP6 dataset [51,52]. CMIP6 is the latest scenario, published in the AR6 of the IPCC in 2021. The pattern of evolution and characteristics adaptation of the previous CMIP, such as CMIP5, continues in CMIP6; nevertheless, CMIP6 evolves from centralized activity to a federated activity with many individual Model Intercomparison Projects (MIPs). CMIP6 has more components and higher spatial resolution [53]. Previous studies using the DSSAT model have yet to investigate the combined effects of multiple climate variables, such as temperature, CO_2_, and precipitation, on potato yields.

Therefore, the objectives of this research are:

- To collect potato management, soil, weather, and future climate scenario data;
- To calibrate and validate the DSSAT model for a better performance;
- To assess the impacts of climate change on potato yields in PEI.

Specifically, we will collect the required data to set up the DSSAT model, calibrate, and validate the model. Further, we will use the CMIP6 data from seven global climate models (GCMs), including CanESM5, FGOAL-G3, GFDL-ESM4, MIROC6, MRI-ESM2, IPSL-CM6A-LR, and EC-Earth3-Veg, under five shared socioeconomic pathways (SSPs), including SSP1–1.9, SSP1–2.6, SSP2–4.5, SSP3–7.0, and SSP5–8.5. We will use the CMIP6 data to drive the DSSAT model to evaluate the combined effects of changing climatic conditions, including maximum temperature (T_max_), minimum temperature (T_min_), precipitation, and CO_2_ concentration in the atmosphere on potato yields in PEI. The results from this research can help potato farmers and stakeholders in PEI understand the ongoing and future challenges that the changing climate will bring to the local potato industries. Furthermore, this study can provide a scientific base for policymakers to develop climate change mitigation and adaptation measures to support sustainable production in the PEI potato sector.

## 2. Data and Methods

This study uses methods and procedures according to DSSAT (2022) [54,55,56,57,58]. Specifically, we first collect the required input data, referred to as minimum dataset (MDS), for model calibration and validation. The calibration and validation process follow subsequent stages as data collection (observation or measurement) of the experimental data (planting, maturity dates, and tuber yields), calibration of the model using experimental data; sensitivity analysis; validation of the model; assessment of the possibilities and limitations (simulation of potential yields); and the climate change impacts on the yields.

### 2.1. Study Area

**Figure 1:**
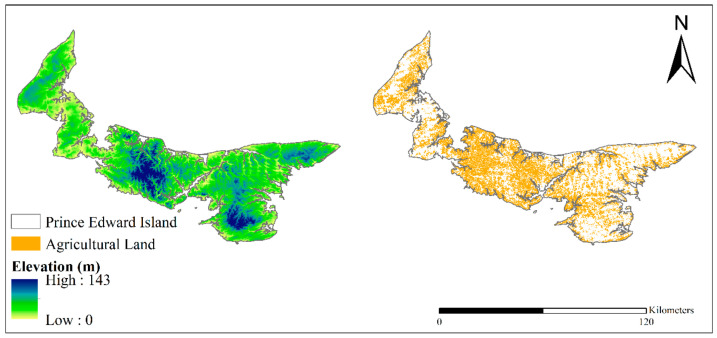
Elevation and agricultural lands in PEI.

This study focuses on the smallest province in Canada, PEI (Figure 1), popularly called the Island, located off the eastern coast of Canada. PEI is less populated and considered part of Atlantic Canada [10,59].

**Figure 2:**
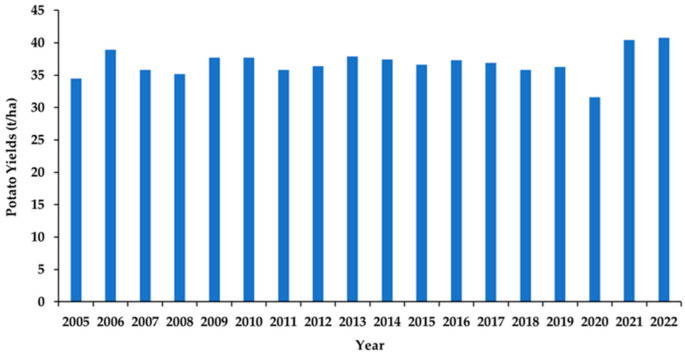
Annual average potato yields in PEI from 2005 to 2022.

PEI is a province that produces and exports a significant quantity of potatoes among other Canadian provinces. As of 1 October 2022, the population of PEI was estimated to be 172,707. The province lies between 46 °N to 47 °N latitude and 62 °W to 64 °W longitude with a total land area of 566,560 ha. Farming occurs on about 42.5% of the total land area (240,514 ha) and ultimately supports the Island’s economy, with about 35,378 ha used to cultivate potatoes. The Island produces one-fourth of the potatoes produced in Canada [9,13,60,61]. PEI potatoes are dominant in Canada and contribute significantly to the agricultural economy of PEI. Although potatoes cultivated in PEI have a high nutritional value and quality, according to FAO comparison of potato production, there has been a decline in Canadian potato production since 2017 [8]; specifically, the annual potato yields in PEI [9,62] have fluctuated over the years (Figure 2). Climate change is suggested to be responsible for the instability of PEI potato yields; in essence, there is a need to study the impacts of climate change on potato yields scientifically [13,42,50,63].

### 2.2. Data Collection

#### 2.2.1. Weather Data

The experiment involves daily measurements of precipitation, solar radiation, T_max_, and T_min_. Daily weather data were downloaded from the “Environment and Climate Change Canada” website, while solar radiation is from the National Aeronautics and Space Administration’s “NASA-Prediction of Worldwide Energy Resource” website [64,65]. The DSSAT Weatherman module is set up and run with the weather files.

#### 2.2.2. Soil Data

Quantitative information on soil (Orthic Humo-Ferric Podzol class) texture and organic carbon are computed with other parameters such as the drained upper limit, saturated water content, saturated hydraulic conductivity, root growth factor, and lower limit using the Sbuild module, a soil parameter estimation tool in the DSSAT suite (Table 1), and used to set up the DSSAT soil module. Customized soil files by depth, between 10–90 cm, are from the literature of a PEI study [63].

#### 2.2.3. Crop Management and Experimental Data

The study follows standard agronomic and management practices to set up the Xbuild and ATCreate modules. Crop management and experimental data are collected through a survey with assistance from the PEI Potato Board and local farmers (see the survey form in Supplementary Information S1). Other required crop management and experimental data are collected from the PEI Potato Board [14]. The collected data are used to simulate PEI’s potato yields under rainfed conditions. The primary management practices are shown in Table 2.

#### 2.2.4. Future Climate Scenarios

In our impacts assessment study, we use one historical period (1995 to 2014) as the baseline and three future periods, the 2050s (2045–2055), 2070s (2065–2075), 2090s (2085–2095) under five SSPs (including SSP1–1.9, SSP1–2.6, SSP2–4.5, SSP3–7.0, and SSP5–8.5). We use GCMs data, including CanESM5, FGOAL-G3, GFDL-ESM4, MIROC6, MRI-ESM2, IPSL-CM6A-LR, and EC-Earth3-Veg (Table 3), from CMIP6 developed by IPCC, considering the resolutions for each GCMs accordingly in the study. The data are from the World Bank Climate Knowledge portal and IPCC Our World in Data sites [51,52].

**Figure 3:**
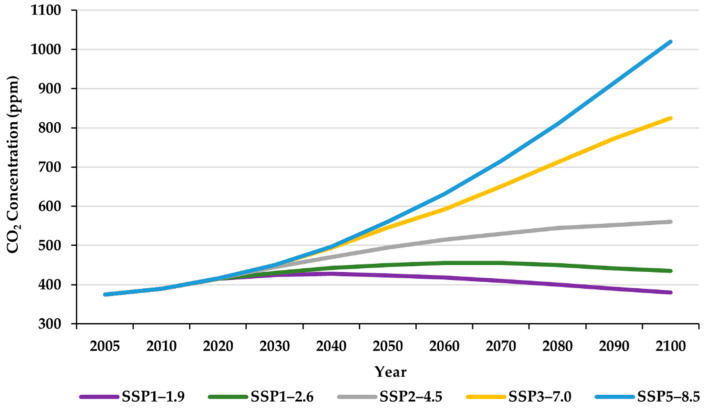
CO_2_ concentrations under CMIP6 SSP emission scenarios.

SSPs describe the possible range of future climates based on human development, economy, environmental action, atmospheric CO_2_ concentration, and sustainability as essential features. Each scenario depicts climate information with specified conditions, and SSP5 represents the worst-case scenario. SSP1 reflects a steadily shifting world, sustainable with regular challenges to mitigation and adaptation. SSP2 is with medium challenges to mitigation and adaptation, representing the middle of the road. SSP3 has mitigation and adaptation with high challenges, leading to a rivalry condition in regions. SSP5 exhibits frequent challenges to adaptation but high challenges to mitigation in a fossil-fueled development era [67,68,69]. This study considered five greenhouse gas (GHG) emission scenarios, including SSP1–1.9, SSP1–2.6, SSP2–4.5, SSP3–7.0, and SSP5–8.5 (Figure 3).

### 2.3. DSSAT Potato Model

**Figure 4:**
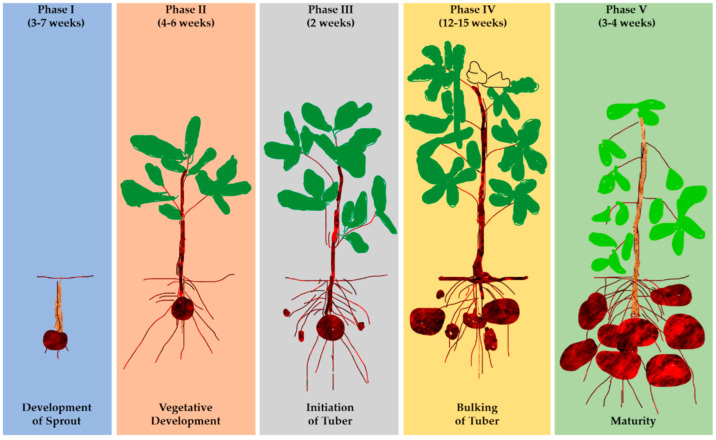
The development stages of potatoes.

The DSSAT model is a collection of independent application programs that operate together, developed by the International Benchmark Sites Network for Agrotechnology Transfer (IBSNAT). There are 42 crop modules with tools to expedite the efficient use of the model [56,57,58]. The tools include a program database for weather, soil, crop management, experimental data, and applications. The CSM assesses crop growth and development as a function of the soil–plant–atmosphere dynamics related to soil, weather, crop experiment, management practices, genotypes, water, and nitrogen dynamics in the databases [58,70,71]. The DSSAT model comprises modules, databases, and applications controlled by software to aid in selecting and comparing alternatives to predict results [71]. It archives and supplies the data to the models for simulating the various kinds of experimental situations and assessing the risks or simulating yields on a long-term basis [58,72]. Simulation of Underground Bulking Storage Organ component (SUBSTOR) is a member of the sixteen computer software application programs that use Formula Translation (FORTRAN) language embedded within the DSSAT model. It assesses the potato’s phenological effect, yield accumulation, and biomass in response to environmental factors [73,74,75]. It is basically used for various agroclimatic conditions and comprises modules that are used to input data, mathematical calculations of the process of growth and development, and, finally, interpretation of the potato simulation outputs [3,56]. The model considers several functions simultaneously to produce the actual structure of the soil–crop–atmospheric dynamic at different potato growth stages [75,76,77]. Potato development (Figure 4) occurs in various stages: sprout elongation, emergence, tuber initiation, bulking, and maturity [78].

SUBSTOR uses five genotype coefficients to define a potato cultivar’s growth and development, which determines how the cultivar reacts to climatic conditions. The genetic coefficients include (1) G2—the rate of leaf area expansion, (2) G3—the rate of potential tuber growth, (3) PD—an index that suppresses tuber growth (dimensionless), (4) P2—sensitivity of tuber initiation to photoperiod (dimensionless), and (5) TC—tuber initiation upper critical temperature (Table 4). The varying genetic coefficients affect potato biomass accumulation [33,46,73,79,80,81]. SUBSTOR is partitioned into sub-sections simultaneously, modeling soil water, nitrogen balances, phenological development, partitioning, and biomass formation of potato crops, producing a real plant–soil–atmospheric system description [58,70]. TC and P2 are vital parameters at the tuber initiation stage; initiation and bulking are inhibited when the TC is exceeded, and a particular cultivar is less sensitive to long photoperiods the closer P2 tends towards 0. G2, G3, and PD influence biomass accumulation [74].

### 2.4. Model Calibration and Validation

The DSSAT model estimates potato yields in dry tuber weight using five genetic coefficients describing crop growth and development processes. Under different weather, soil, and management conditions, the potato coefficients aid the model in simulating the performance of the genotypes. An accurate simulation of tuber yields requires the correct genetic coefficients. We use the genotype coefficient calculator (GENCALC) module in the DSSAT model to calibrate model parameters in Table 4. Cultivar parameters are varied over a wide range to capture the behavior of the crop across a wide genetic range. Each parameter varies while holding the other four parameters constant at their calibration values. The potential of using a physically based (dynamic) crop simulation model (i.e., model performance) is evaluated by comparing the aggregated and reported tuber yields. The coefficient of determination (i.e., *R*^2^), Nash–Sutcliffe efficiency (i.e., *NSE*), and index of agreement (i.e., *d*-stat) are used to ascertain the agreement between the observed and simulated values. *R*^2^ ranges from 0 to 1; the closer the value to 1, the better the agreement between the observed yield and the simulated yield, and a value of 1 shows a perfect correlation, i.e., 0 ≤ *R*^2^ ≤ 1 [82]. *R*^2^ is calculated using the following equation.

(1) $$R^{2}=1-\frac{SSE}{SST}$$

*R*^2^ is the coefficient of determination, *SSE* is the sum of squared error, and *SST* is the sum of squares total. Equation (1) is used to confirm the *R*^2^ generated by the model. Sensitivity analyses explore the variation of the genetic coefficient on potato yields to validate the model. *NSE* ranges from −∞ to 1, where *NSE* is considered good between 0.75 and 1, satisfactory between 0.36 and 0.75, and unacceptable when below 0.36, i.e., 0.36 ≤ *NSE* ≤ 1 [83,84]. *NSE* is calculated using the following equation.

(2) $$NSE=1-\frac{\left(\sum_{i=1}^{n}{\left(X_{i}-Y_{i}\right)}^{2}\right)}{\left(\sum_{i=1}^{n}{\left(X_{i}-\bar{X}\right)}^{2}\right)}$$

NSE is the Nash–Sutcliffe efficiency, *X_i_* is the observed value, *Y_i_* is the predicted value, $\bar{X}$ is the observed mean, and n is the number of observations. Equation (2) is used to calculate NSE. The d-stat value ranges from 0 to 1, where the closer the value to 1, the better the agreement between the observed yield and the simulated yield, where *d* = 1 shows a perfect agreement, i.e., 0 ≤ *d* ≤ 1 [81,83]. The *d*-stat is calculated using the following equation.

(3) $$\;d=1-\frac{\sum_{i=1}^{n}{\left(Y_{i}-X_{i}\right)}^{2}}{\sum_{i=n}^{n}{\left(\left|Y_{i}-\bar{Y}\right|+\left|X_{i}-\bar{X}\right|\right)}^{2}}$$

The *d*-stat is the index of agreement, *X_i_* and *Y_i_* are observed and simulated yield values, respectively, $\bar{X}$ and $\bar{Y}$ are the average observed and simulated yield values, respectively, and n is the observation numbers. Equation (3) is used to calculate the *d*-stat.

### 2.5. Measuring Climate Change Impacts on Potato Yields

The DSSAT model uses CMIP6 data to assess the impacts of changing climate on potato yields in PEI, with the coefficients validated in this study. The potato yields estimated under the baseline are compared to those under emission scenarios between 2045 and 2095, and the average of the seven GCMs was considered. The planting and harvest dates are fixed each year with rainfed conditions, considering the planting date that reports the most significant tuber yields. The entire PEI potato crop cycle is set to 130 days from planting to harvesting. The baseline (1995–2014) assessed the historical yields, and the assessment under each of the five emission classes captures the effect of climate variability on future yields. The percentage yield changes under future climates were evaluated by the average yield changes from the GCMs compared to the baseline yields. Under this approach, the changes in potato yield are calculated using the following equation:

(4) $$\mathrm{Change}\;\mathrm{in}\;\mathrm{Yield}\;\left(\%\right)=\left(\frac{\mathrm{Future}\;\mathrm{Yield}-\mathrm{Baseline}\;\mathrm{Yield}}{\mathrm{Baseline}\;\mathrm{Yield}}\right)\times 100\%$$

Emission scenarios reveal a future decline in potato yields compared to the baseline.

## 3. Results

The results for calibration, validation, and assessment of the impacts of climate change on potato yields are summarized and discussed below.

### 3.1. Model Calibration and Validation

The DSSAT model used PEI conditions (i.e., soil, weather, and crop management practices data) to successfully perform the genetic coefficient estimation (i.e., calibration and validation) by running the sub-model (GENCALC) to predict rainfed tuber yields for potato variety RB. Calibration and validation are vital for improving model performance and involve comparing field measurements (data) with the model outputs. In this study, the model was calibrated using ten years of “2000–2009” (Table 5) data and validated with eight years of “2010–2017” (Table 6) data in order to compare the observed to the simulated yield. The simulated data are generated through SUBSTOR (potato module) under the DSSAT model interface and compared to the observed yield. The observed yields are island-wide harvested potato yields from potato farms in PEI [50,85].

**Figure 5:**
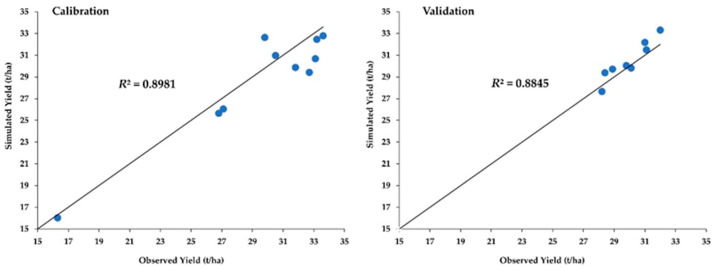
The results for model calibration and validation.

The *R*^2^ with the intercept set to zero were 0.898 and 0.885 for calibration and validation, respectively (Figure 5), indicating a better and closer correlation. The results indicate a significant correlation between observed and simulated yields (i.e., the observed tuber yields corresponded well with the simulated tuber yields), representing a good performance. Additionally, the *NSE* and *d*-stat are 0.87 and 0.92, respectively, indicating a good correlation between observed yield and simulated yield.

The derived genetic coefficients are used to quantify the development responses of rainfed tuber yields of RB to the changing climate. The mean observed and simulated yields for calibration are 29.5 t/ha and 28.7 t/ha, respectively, and for validation, they are 30.0 t/ha and 30.5 t/ha, respectively. The validated calibration shows that the DSSAT model can accurately assess potato yields under different management conditions in different climatic regions.

### 3.2. Impacts of Future Climate Change on Potato Yields

**Figure 6:**
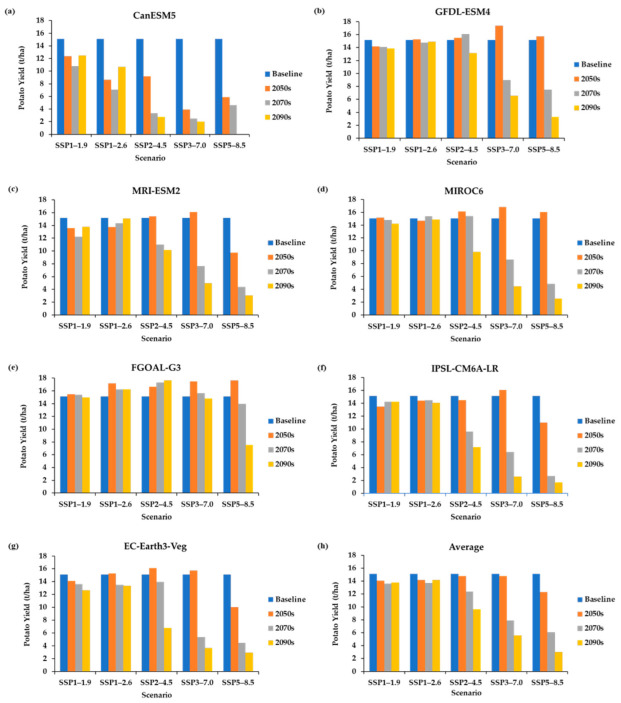
PEI’s future potato yields under CMIP6 scenarios. (**a**) CanESM5, (**b**) GFDL-ESM4, (**c**) MRI-ESM2, (**d**) MIROC6, (**e**) FGOAL-G3, (**f**) IPSL-CM6A-LR, (**g**) EC-Earth3-Veg, and (**h**) multi-model average.

The DSSAT model assesses the impacts of climate change on potato yields, showing that the high-emission scenario could result in a significant decline. Future potato yields were assessed using CMIP6 data for the 2050s, 2070s, and 2090s compared with the baseline period (1995–2014) for SSP1–1.9, SSP1–2.6, SSP2–4.5, SSP3–7.0, and SSP5–8.5. The yield assessment using T_max_, T_min_, precipitation, and CO_2_ was compared among the generated climate scenarios and the baseline period “1995–2014.” The results indicated that the projections were based on combinations of seven GCMs under five SSP scenarios. Across the seven GCMs, CanESM5 shows the most significant decline, while the least decline is observed in the FGOAL-G3 (Figure 6 and Table 7). Overall, there is a significant decline in high emission scenarios, especially towards the end of the century when the average of the seven GCMs projection is considered.

There is a considerable variation in observed temperature, precipitation, and CO_2_ for future climate scenarios. T_max_, T_min_, precipitation, and CO_2_ are estimated to increase compared to the baseline over the century until the worst emission scenario (SSP5–8.5). Overall average yield decline is expected as simulated by the seven GCMs for the five SSPs, which projects to be significant towards the end of the year. The model average projected that it is likely to have the most significant yield decline under SSP5–8.5, followed by SSP3–7.0 and then SSP2–4.5, with medium to low yield decline in SSP1–1.9 and SSP1–2.6.

Extensively, the average potato yields in the future, under SSP1–1.9, will likely decline from 14.0 t/ha in the 2050s to 13.6 t/ha in the 2070s and 13.7 t/ha in the 2090s. Under SSP1–2.6, the yields are expected to decline from 14.2 t/ha in the 2050s to 13.7 t/ha in the 2070s and increase to 14.2 t/ha in the 2090s. In addition, under SSP2–4.5, the yields suggest a decline from 14.8 t/ha in the 2050s to 12.4 t/ha in the 2070s and 9.6 t/ha in the 2090s. Furthermore, yields under SSP3–7.0 are expected to decline from 14.8 t/ha in the 2050s to 7.9 t/ha in the 2070s and 5.6 t/ha in the 2090s. The most significant yield decline is expected under SSP5–8.5, from 12.3 t/ha in the 2050s to 6.1 t/ha in the 2070s and 3.0 t/ha in the 2090s.

**Figure 7:**
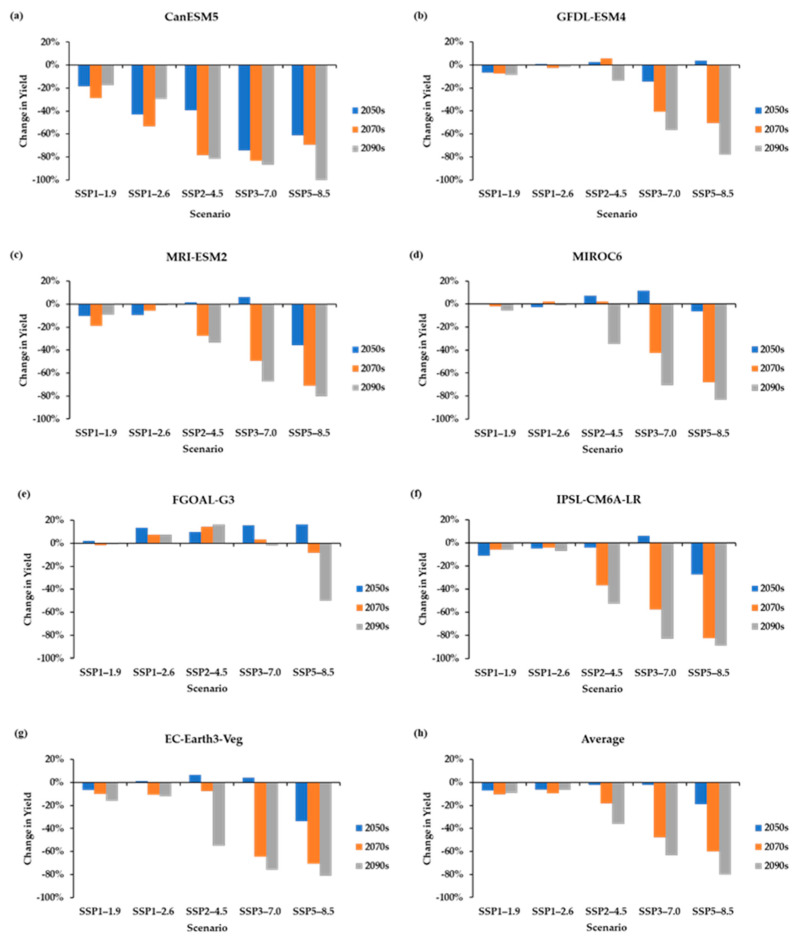
Percentage change in PEI’s potato yields under CMIP6 scenarios. (**a**) CanESM5, (**b**) GFDL-ESM4, (**c**) MRI-ESM2, (**d**) MIROC6, (**e**) FGOAL-G3, (**f**) IPSL-CM6A-LR, (**g**) EC-Earth3-Veg, and (**h**) multi-model average.

Compared to the baseline, yields under the SSP1–1.9 suggests a decline of 7.2% in the 2050s, 10.2% in the 2070s, and 9.2% in the 2090s. Under the SSP1–2.6, the yields are expected to decline by 6.4% in the 2050s, 9.6% in the 2070s, and 6.4% in the 2090s. Under the SSP2–4.5, the yields suggest a decline of 2.2% in the 2050s, 18.3% in the 2070s, and 36.4% in the 2090s. Yield decline of 2.3%, 47.8%, and 63.2% is likely under SSP3–7.0 in the 2050s, 2070s, and 2090s, respectively, while a decline of 18.8%, 60.0%, and 80.1% are expected in the 2050s, 2070s, and 2090s under SSP5–8.5 (see Figure 7 and Table 8).

However, the results were inconsistent between the different GCMs; there were mixed results, including decreases (red cells in Table 8) and increases (green cells in Table 8) in yields under the periods and SSPs.

## 4. Discussions

The current study showed that potato yields are expected to decrease in PEI toward the end of the century when we consider the combined effect of T_max_, T_min_, precipitation, and CO_2_. The seven GCMs projected increased temperature in the future, i.e., in the 2050s–2090s, T_max_ and T_min_ are expected to increase by 1.2 °C to 5.6 °C and 1.4 °C to 6.1 °C, respectively, depending on the climate scenario and period. CO_2_ increased by 2.4% to 140.9%, while precipitation increased by 3.4% to 12.8% compared to the baseline values. There is instability in precipitation patterns across the scenarios and period; hence, the yield decline can be attributed to precipitation variation because the significant potato yield changes correlate with precipitation changes.

Our study result showed that future yields decrease under rainfed conditions, compared to Brassard and Singh’s (2007) [47] studies, which reported a decrease in future potato yields in Quebec. In addition, Vashisht et al.’s (2015) [83] studies showed that future potato production under rainfed conditions in Minnesota, US, is projected to decrease due to climate change. Nevertheless, other studies showed that climate change might increase future potato yields. For instance, Tooley et al. (2021) [86] reported increased future potato yields in Maine, US, due to climate change, while some studies projected increased future potato yields under rainfed conditions compared to irrigated conditions [87,88]. Furthermore, Tubiello F. N. et al. (2002) [89] projected that climate change would increase future potato yield in the northern United States of America (US) while it will be reduced in the southern areas of the US. The observed variations in the projected future potato yields are due to differences in geographical area and management practices. The variation could also be caused by the differences in the GCMs used [74]. Our study suggests that the potato yield decrease correlates with an increase in future temperature and CO_2_ concentration with varying precipitation patterns. Overall, our results indicate the potential negative impacts of climate change on future potato yields under rainfed conditions in PEI. It is worth mentioning that the crop yields determinant is not only limited to temperature, precipitation, and CO_2_ but also depends on other factors, such as pests, soil salinity, and other parameters which play an essential role in crop growth processes and the harvested yields but are not included in our study’s scope [26,29,30,90,91]. Although temperature and CO_2_ changes influence the yields, their reaction with precipitation significantly influences tuber yields under rainfed conditions in PEI. The amount of precipitation and distribution within a specified temperature range drives potato development. Precipitation affected the yields simulated, which mostly declined from decreased precipitation with some compensation through elevated atmospheric CO_2_. Our results suggest that future potato yields are expected to decrease in PEI, which could be attributed to the future drought effect under rainfed potato production systems. Considering that PEI potato cultivation is significantly rainfed [13,92], and since the decrease in precipitation decreases our simulated yield, we attributed the decline in potato yields to the drought effect, which could be compensated through supplemental irrigation. This study is a foundation to examine further and ascertain proper adaptation strategies to increase potato yields, which can be recommended to farmers. Ultimately, this study methodology can be applied to assess the impacts of climate change on potato yields in any geographical region worldwide.

## 5. Conclusions

In this study, we used the DSSAT model to assess the potential impacts of future climate change on potato yields in PEI. In particular, we used the IPCC CMIP6 data under five GHG emission scenarios, SSP1–1.9, SSP1–2.6, SSP2–4.5, SSP3–7.0, and SSP5–8.5, to assess the effects of climate change on potato yields in PEI. GCMs (CanESM5, FGOAL-G3, GFDL-ESM4, MIROC6, MRI-ESM2, IPSL-CM6A-LR, and EC-Earth3-Veg) were used to generate emission scenarios for the study. The assessment evaluates the combined impacts of climate variables, T_max_, T_min_, precipitation, and CO_2,_ on potato yields.

This study calibrates and validates the DSSAT model using dry tuber weight as the parameter to evaluate the model’s performance. The observed and simulated values were in close agreement and fell within the statistical significance limit. Using the average of the GCMs, the potato yields suggested a gradual decline under SSP1–1.9 and SSP1–2.6, with a distinct decline under SSP2–4.5. The most significant decline is expected under high-emission scenarios SSP3–7.0 and SSP5–8.5. The reduction is expected to be enormous towards the end of the century, indicating significant negative impacts on the yields due to climate change. Adapting to climate change’s impacts requires exploring various strategies to guarantee food security. These strategies are crucial to improve crop and soil management and enhancing potato production. The results from this study can provide farmers and policymakers with a scientific basis to develop coping mechanisms for climate change impacts which can be adopted for optimal and quality potato yields to ensure food security.

## Figures and Tables

**Table 1:** Soil properties for PEI.

Depth (cm)	Clay (%)	Silt (%)	Org. C. (%)	BD (Mg·m^−3^)	pH	DUL	SWC	SHC (cm·h)	RGF	LL
0–10	8	36	1.9	1.33	6.1	0.272	0.459	2.59	1	0.118
10–20	9	38	1.9	1.39	6.1	0.282	0.437	2.59	1	0.123
20–30	9	35	1.6	1.39	6.2	2.260	0.439	2.59	0.607	0.115
30–40	9	37	1.2	1.54	6.2	0.244	0.388	2.59	0.497	0.104
40–50	11	36	0.5	1.60	6.2	0.217	0.372	2.59	0.407	0.095
50–60	13	34	0.2	1.60	6.3	0.209	0.375	2.59	0.333	0.096
60–70	14	34	0.2	1.79	6.3	0.214	0.306	2.59	0.273	0.101
70–80	13	35	0.1	1.79	6.3	0.206	0.307	2.59	0.223	0.094
80–90	14	35	0.1	1.79	6.4	0.211	0.307	1.32	0.183	0.099

Note: Org. C—Organic Carbon, BD—Bulk Density, DUL—Drained Upper Limit, SWC—Saturated Water Content, SHC—Saturated Hydraulic Conduct, RGF—Root Growth Factor, and LL—Lower Limit.

**Table 2:** Key information about the potato management practices in PEI.

Crop	Potato
Cultivar	Russet Burbank *
Planting month	May
Planting method	Dry Seed
Planting depth	6.5 inches
Planting distribution	Rows
Row spacing	14 inches
Fertilizer	Ammonium Nitrate
Tillage	Mouldboard Plough
Irrigation	Not Irrigated
Harvest month	October
Length of the growing season	130 days
Other practices (e.g., tillage type, depth, and date, initial condition, fertilizer depth, and application date)	Observed according to the recommended practices by the PEI potato board and farmers

* In this study, we use the potato variety Russet Burbank (RB) because it is the most common (about 90%) among the potato varieties in PEI [66]. Additionally, the survey results we received in this study are mostly about RB, and no sufficient data are collected for other varieties in PEI. Note that no supplemental irrigation plan was implemented in PEI for the study period of 1995–2014.

**Table 3:** List of global climate models used in this study.

Full Name of GCM	Abbreviation	Institute
The Canadian Earth System Model version 5	CanESM5	Canadian Centre for Climate Modelling and Analysis, Canada https://www.canada.ca/en/environment-climate-change/services/science-technology/centres/british-columbia.html#cccma (accessed on 12 November 2022)
Geophysical Fluid Dynamics Laboratory Earth System Model version 4	GFDL-ESM4	Geophysical Fluid Dynamics Laboratory, United States of America https://www.gfdl.noaa.gov/ (accessed on 12 November 2022)
The Meteorological Research Institute Earth System Model version 2	MRI-ESM2	The Meteorological Research Institute, Japan https://www.mri-jma.go.jp/index_en.html (accessed on 12 November 2022)
Model for Interdisciplinary Research on Climate version 6	MIROC6	Division of Climate System Research, Japan https://ccsr.aori.u-tokyo.ac.jp/index-e.html (accessed on 12 November 2022)
Flexible Global Ocean-Atmosphere-Land System Model: Grid-Point version 3	FGOAL-G3	The State Key Laboratory of Numerical Modeling for Atmospheric Sciences and Geophysical Fluid Dynamics- Institute of Atmospheric Physics, China http://english.iap.cas.cn/rh/rd/200906/t20090626_9069.html (accessed on 12 November 2022)
The Institute Pierre-Simon Laplace Climate Model	IPSL-CM6A-LR	The Institute Pierre-Simon Laplace Climate Modelling Center, France https://cmc.ipsl.fr/ (accessed on 12 November 2022)
European Community Earth 3 with interactive vegetation module at low resolution	EC-Earth3-Veg	12 European Countries https://ec-earth.org (accessed on 12 November 2022)

**Table 4:** Calibrated parameters in the DSSAT model.

Symbol	Parameter	Range	Iteration Interval	Calibrated Genetic Coefficient	Units
G2	Leaf area expansion rate after tuber initiation	900–2100	5	2100	cm^2^·m^−2^·day^−1^
G3	Potential tuber growth rate	21–26	0.02	21	g·m^−2^·day^−1^
PD	Suppression of tuber growth following tuber induction	0.5–1.0	0.01	0.500	relative index
P2	Tuber initiation sensitivity to long photoperiods	0.3–0.9	0.01	0.586	relative index
TC	The upper critical temperature for tuber initiation	5–22	0.02	22	°C

**Table 5:** Observed and simulated yield (2000–2009).

Year	Observed (t/ha)	Simulated (t/ha)
2000	29.8	32.6
2001	16.3	16.0
2002	32.7	29.4
2003	30.5	31.0
2004	33.1	30.7
2005	31.8	29.9
2006	33.2	32.5
2007	33.6	32.8
2008	26.8	25.7
2009	27.1	26.1

**Table 6:** Observed and simulated yield (2010–2017).

Year	Observed (t/ha)	Simulated (t/ha)
2010	29.8	30.1
2011	28.9	29.7
2012	28.2	27.7
2013	28.4	29.4
2014	31.1	31.5
2015	31.0	32.2
2016	32.0	33.3
2017	30.1	29.8

**Table 7:** PEI’s future potato yields (units: t/ha) under SSP scenarios.

GCM	Period	SSP Scenario				
		SSP1–1.9	SSP1–2.6	SSP−4.5	SSP3–7.0	SSP5–8.5
CanESM5	Baseline	15.1				
	2050s	12.3	8.6	9.2	3.9	5.9
	2070s	10.8	7.0	3.3	2.5	4.6
	2090s	12.4	10.7	2.8	2.0	0
GFDL-ESM4	Baseline	15.2				
	2050s	14.1	15.3	15.5	17.4	15.7
	2070s	14.1	14.8	16.0	9.0	7.5
	2090s	13.9	14.9	13.1	6.6	3.3
MRI-ESM2	Baseline	15.2				
	2050s	13.6	13.8	15.4	16.1	9.8
	2070s	12.3	143	11.0	7.7	4.4
	2090s	13.8	15.1	10.1	5.0	3.1
MIROC6	Baseline	15.1				
	2050s	15.2	14.7	16.2	16,8	16.0
	2070s	14.8	15.4	15.4	8.6	4.8
	2090s	14.2	14.9	9.8	4.4	2.6
FGOAL-G3	Baseline	15.1				
	2050s	15.5	17.2	16.6	17.5	17.6
	2070s	15.4	16.3	17.3	15.6	13.9
	2090s	15.0	16.2	17.6	14.8	7.6
IPSL-CM6A-LR	Baseline	15.1				
	2050s	13.5	14.4	14.5	16.0	11.0
	2070s	14.2	14.5	9.6	6.4	2.7
	2090s	14.3	14.1	7.2	2.6	1.7
EC-Earth3-Veg	Baseline	15.1				
	2050s	14.1	15.3	16.1	15.7	10.0
	2070s	13.6	13.5	14.0	5.4	4.5
	2090s	12.7	13.3	6.8	3.7	2.9
Model Average	Baseline	15.1				
	2050s	14.0	14.2	14.8	14.8	12.3
	2070s	13.6	13.7	12.4	7.9	6.1
	2090s	13.7	14.2	9.6	5.6	3.0

**Table 8:** Percentage change in PEI’s potato yields under SSP scenarios.

GCM	Period	SSP Scenario				
		SSP1–1.9	SSP1–2.6	SSP−4.5	SSP3–7.0	SSP5–8.5
CanESM5	2050s	−18.3%	−43.0%	−39.2%	−74.3%	−60.9%
	2070s	−28.8%	−53.5%	−78.2%	−83.4%	−69.5%
	2090s	−17.7%	−29.3%	−81.6%	−86.6%	−100%
GFDL-ESM4	2050s	−6.8%	+0.8%	+2.4%	−14.6%	+3.6%
	2070s	−7.5%	−2.7%	+5.7%	−40.8%	−50.6%
	2090s	−8.6%	−1.8%	−13.5%	−56.8%	−78.1%
MRI-ESM2	2050s	−10.4%	−9.4%	+1.5%	+6.2%	−35.6%
	2070s	−19.1%	−5.7%	−27.7%	−49.5%	−71.1%
	2090s	−9.3%	−0.7%	−33.3%	−67.3%	−79.9%
MIROC6	2050s	+0.6%	−2.7%	+7.2%	+11.8%	−6.3%
	2070s	−2.0%	+2.0%	+2.2%	−42.7%	−67.9%
	2090s	−5.5%	−1.3%	−34.9%	−70.5%	−83.1%
FGOAL-G3	2050s	+2.0%	+13.5%	+9.9%	+15.5%	+16.4%
	2070s	−1.8%	+7.5%	+14.3%	+3.2%	−8.1%
	2090s	−1.1%	+7.3%	+16.4%	−2.2%	−50.1%
IPSL-CM6A-LR	2050s	−10.8%	−4.9%	−4.2%	+6.1%	−27.3%
	2070s	−5.8%	−4.1%	−36.8%	−57.4%	−82.4%
	2090s	−5.7%	−6.9%	−52.6%	−83.0%	−88.8%
EC-Earth3-Veg	2050s	−6.7%	+1.2%	+6.7%	+4.1%	−33.8%
	2070s	−9.8%	−10.8%	−7.4%	−64.3%	−70.5%
	2090s	−16.2%	−11.9%	−55.2%	−75.9%	−80.7%
Model Average	2050s	−7.2%	−6.4%	−2.2%	−2.3%	−18.8%
	2070s	−10.2%	−9.6%	−18.3%	−47.8%	−60.0%
	2090s	−9.2%	−6.4%	−36.4%	−63.2%	−80.1%

Note that the red cells showed percentage decrease in PEI potato yields while the green cells showed percentage increase in PEI potato yields, across the periods and GCMs under SSP scenarios.

## Data Availability

Data is contained within the article or Supplementary Material.

## Supplementary Materials

The following supporting information can be downloaded at: https://www.mdpi.com/article/10.3390/foods12061176/s1, Supplementary Information S1: Survey Form on PEI Potato Farming Practices.

## References

[1] Celis-Gamboa B.C. (2002). The Life Cycle of the Potato (Solanum tuberosum L.): From Crop Physiology to Genetics.

[2] Zaheer K., Akhtar M.H. (2014). Recent Advances in Potato Production, Usage, Nutrition—A Review. Crit. Rev. Food Sci. Nutr..

[3] Kumar Y., Singh R., Kumar A. (2021). Performance of SUBSTOR Model on Growth and Yield of Potato Varieties under Different Planting Dates in a Sub-Tropical Environment. J. Agrometeorol..

[4] FAOSTAT Potato Production. https://www.fao.org/faostat/en/#data/QCL.

[5] Jennings S.A., Koehler A.K., Nicklin K.J., Deva C., Sait S.M., Challinor A.J. (2020). Global Potato Yields Increase Under Climate Change with Adaptation and CO_2_ Fertilisation. Front. Sustain. Food Syst..

[6] Ahmadu T., Abdullahi A., Ahmad K., Ahmadu T., Abdullahi A., Ahmad K., Yildiz M., Ozgen Y. (2021). The Role of Crop Protection in Sustainable Potato (*Solanum Tuberosum* L.) Production to Alleviate Global Starvation Problem: An Overview. Solanum Tuberosum—A Promising Crop for Starvation Problem.

[7] Devaux A., Kromann P., Ortiz O. (2014). Potatoes for Sustainable Global Food Security. Potato Res..

[8] FAOSTAT Canada Potato Production. https://www.fao.org/faostat/en/#compare.

[9] Statistics Canada Table 32-10-0358-01 Area, Production and Farm Value of Potatoes. https://www150.statcan.gc.ca/t1/tbl1/en/tv.action?pid=3210035801&pickMembers%5B0%5D=1.3&cubeTimeFrame.startYear=2005&cubeTimeFrame.endYear=2022&referencePeriods=20050101%2C20220101.

[10] Statistics Canada Table 17-10-0009-01 Population Estimates, Quarterly. https://www150.statcan.gc.ca/t1/tbl1/en/tv.action?pid=1710000901.

[11] Statistics Canada Canadian Potato Production. https://www150.statcan.gc.ca/n1/daily-quotidien/221207/dq221207d-eng.htm.

[12] Danielescu S., MacQuarrie K.T.B., Zebarth B., Nyiraneza J., Grimmett M., Levesque M. (2022). Crop Water Deficit and Supplemental Irrigation Requirements for Potato Production in a Temperate Humid Region (Prince Edward Island, Canada). Water.

[13] Maqsood J., Farooque A.A., Wang X., Abbas F., Acharya B., Afzaal H. (2020). Contribution of Climate Extremes to Variation in Potato Tuber Yield in Prince Edward Island. Sustainability.

[14] PEI Potato Board Potatoes from Prince Edward Island. https://www.peipotato.org/pei-potato-industry/about-us.

[15] Beals K.A. (2019). Potatoes, Nutrition and Health. Am. J. Potato Res..

[16] Górska-Warsewicz H., Rejman K., Kaczorowska J., Laskowski W. (2021). Vegetables, Potatoes and Their Products as Sources of Energy and Nutrients to the Average Diet in Poland. Int. J. Environ. Res. Public Health.

[17] Navarre D.A., Goyer A., Shakya R. (2009). Nutritional Value of Potatoes: Vitamin, Phytonutrient, and Mineral Content. Advances in Potato Chemistry and Technology.

[18] Devaux A., Goffart J.P., Petsakos A., Kromann P., Gatto M., Okello J., Suarez V., Hareau G. (2019). Global Food Security, Contributions from Sustainable Potato Agri-Food Systems. Potato Crop: Its Agricultural, Nutritional and Social Contribution to Humankind.

[19] Agriculture and Agri-Food Canada Potato Market Information Review 2020–2021. https://agriculture.canada.ca/sites/default/files/documents/2022-02/potato_market_review_revue_marche_pomme_terre_2020-eng.pdf.

[20] Quiroz R., Ramírez D.A., Kroschel J., Andrade-Piedra J., Barreda C., Condori B., Mares V., Monneveux P., Perez W. (2018). Impact of Climate Change on the Potato Crop and Biodiversity in Its Center of Origin. Open Agric..

[21] Dreyer H. (2018). Towards Sustainable Potato Production: Partnering to Support Family Farmers in Africa. Potato Res..

[22] United Nations THE 17 GOALS | Sustainable Development Goals. https://sdgs.un.org/goals.

[23] Raymundo R., Asseng S., Cammarano D., Quiroz R. (2014). Potato, Sweet Potato, and Yam Models for Climate Change: A Review. Field Crops Res..

[24] Aien A., Chaturvedi A.K., Bahuguna R.N., Pal M. (2017). Phenological Sensitivity to High Temperature Stress Determines Dry Matter Partitioning and Yield in Potato. Indian J. Plant Physiol..

[25] Fleisher D.H., Timlin D.J., Reddy V.R. (2006). Temperature Influence on Potato Leaf and Branch Distribution and on Canopy Photosynthetic Rate. Agron. J..

[26] Handayani T., Gilani S.A., Watanabe K.N. (2019). Climatic Changes and Potatoes: How Can We Cope with the Abiotic Stresses?. Breed Sci..

[27] Hijmans R.J. (2003). The Effect of Climate Change on Global Potato Production. Am. J. Potato Res..

[28] Timlin D., Rahman S.M.L., Baker J., Reddy V.R., Fleisher D., Quebedeaux B. (2006). Whole Plant Photosynthesis, Development, and Carbon Partitioning in Potato as a Function of Temperature. Agron. J..

[29] Condori B., Hijmans R.J., Ledent J.F., Quiroz R. (2014). Managing Potato Biodiversity to Cope with Frost Risk in the High Andes: A Modeling Perspective. PLoS ONE.

[30] Hijmans R., Condori B. Estimating the Potential Impact of Frost-Resistant Potato Cultivars in the Altiplano (Peru and Bolivia). Proceedings of the Third International Symposium on Systems Approaches for Agricultural Development.

[31] Naz S., Ahmad S., Abbas G., Fatima Z., Hussain S., Ahmed M., Khan M.A., Khan A., Fahad S., Nasim W. (2022). Modeling the Impact of Climate Warming on Potato Phenology. Eur. J. Agron..

[32] Pulatov B., Linderson M.L., Hall K., Jönsson A.M. (2015). Modeling Climate Change Impact on Potato Crop Phenology, and Risk of Frost Damage and Heat Stress in Northern Europe. Agric. For. Meteorol..

[33] Daccache A., Weatherhead E.K., Stalham M.A., Knox J.W. (2011). Impacts of Climate Change on Irrigated Potato Production in a Humid Climate. Agric. For. Meteorol..

[34] Geddes G. Potatoes and Water Management: From Deluge to Drought-Spud Smart. https://spudsmart.com/potatoes-water-management-deluge-drought/.

[35] CIP International Potato Center Potato Faces Up to Climate Change Challenges-International Potato Center. https://cipotato.org/blog/potato-faces-up-to-climate-change-challenges/.

[36] Kanters Janet Potato Production and Climate Change. https://spudsmart.com/potato-production-climate-change/.

[37] Raymundo R., Asseng S., Robertson R., Petsakos A., Hoogenboom G., Quiroz R., Hareau G., Wolf J. (2018). Climate Change Impact on Global Potato Production. Eur. J. Agron..

[38] Haverkort A.J., Verhagen A. (2008). Climate Change and Its Repercussions for the Potato Supply Chain. Potato Res..

[39] van der Waals J.E., Steyn J.M., Franke A.C., Haverkort A.J. (2016). Grower Perceptions of Biotic and Abiotic Risks of Potato Production in South Africa. Crop Prot..

[40] Srivastava R.K., Talla A., Swain D.K., Panda R.K. (2019). Quantitative Approaches in Adaptation Strategies to Cope with Increased Temperatures Following Climate Change in Potato Crop. Potato Res..

[41] Agriculture and Agri-Food Canada Potato Market Information Review–2019–2020. https://agriculture.canada.ca/en/canadas-agriculture-sectors/horticulture/horticulture-sector-reports/potato-market-information-review-2019-2020.

[42] Azimi M.A., Jiang Y., Meng F.R., Liang K. (2022). Yield Responses of Four Common Potato Cultivars to an Industry Standard and Alternative Rotation in Atlantic Canada. Am. J. Potato Res..

[43] Government of Prince Edward Island (2020). Charlottetown, PE: Strategic Policy and Evaluation Division Department of Agriculture and Land. https://www.princeedwardisland.ca/sites/default/files/publications/af_potato_econ_impact_study.pdf.

[44] Holden N.M., Brereton A.J., Fealy R., Sweeney J. (2003). Possible Change in Irish Climate and Its Impact on Barley and Potato Yields. Agric. For. Meteorol..

[45] Khalid A.M., Hina T., Hameed S., Hamid N.M., Ahmad I., Ur Rehman N., Muhammad A. (2020). Modeling Adaptation Strategies against Climate Change Impacts in Integrated Rice-Wheat Agricultural Production System of Pakistan. Int. J. Environ. Res. Public Health.

[46] Bender F.D., Sentelhas P.C. (2020). Assessment of Regional Climate Change Impacts on Brazilian Potato Tuber Yield. Int. J. Plant Prod..

[47] Brassard J.P., Singh B. (2007). Effects of Climate Change and CO_2_ Increase on Potential Agricultural Production in Southern Quebec, Canada. Clim. Res..

[48] Arunrat N., Sereenonchai S., Chaowiwat W., Wang C. (2022). Climate Change Impact on Major Crop Yield and Water Footprint under CMIP6 Climate Projections in Repeated Drought and Flood Areas in Thailand. Sci. Total Environ..

[49] Zheng E., Qin M., Chen P., Xu T., Zhang Z. (2022). Climate Change Affects the Utilization of Light and Heat Resources in Paddy Field on the Songnen Plain, China. Agriculture.

[50] Jiang Y., Ramsay M., Meng F., Stetson T. (2021). Characterizing Potato Yield Responses to Water Supply in Atlantic Canada’s Humid Climate Using Historical Yield and Weather Data: Implications for Supplemental Irrigation. Agric. Water Manag..

[51] Our World in Data Explorer: IPCC Scenarios. https://ourworldindata.org/explorers/ipcc-scenarios?facet=none&Metric=Greenhouse+gas+emissions&Rate=Total&Region=Global&country=SSP1+-+Baseline~SSP2+-+Baseline~SSP3+-+Baseline~SSP4+-+Baseline~SSP5+-+Baseline.

[52] World Bank Group Download Data | Climate Change Knowledge Portal. https://climateknowledgeportal.worldbank.org/download-data.

[53] Eyring V., Bony S., Meehl G.A., Senior C.A., Stevens B., Stouffer R.J., Taylor K.E. (2016). Overview of the Coupled Model Intercomparison Project Phase 6 (CMIP6) Experimental Design and Organization. Geosci. Model Dev..

[54] DSSAT Cropping Systems Model. https://dssat.net/.

[55] Hoogenboom G., Jones J., Wilkens P., Porter C.H., Batchelor W.D., Hunt L.A., Boote K.J., Singh U., Uryasev O., Bowen W.T. (2004). Decision Support System for Agrotechnology Transfer Version 4.0 [CD-ROM].

[56] Hoogenboom G., Porter C.H., Shelia V., Boote K.J., Singh U., White J.W., Hunt L.A., Ogoshi R., Lizaso J.I., Koo J. Decision Support System for Agrotechnology Transfer (DSSAT) Version 4.7.5. https://dssat.net/2435.

[57] Hoogenboom G., Porter C.H., Boote K.J., Shelia V., Wilkens P.W., Singh U., White J.W., Asseng S., Lizaso J.I., Moreno L.P., Boote K.J. (2019). The DSSAT Crop Modeling Ecosystem. Advances in Crop Modeling for a Sustainable Agriculture.

[58] Jones J.W., Hoogenboom G., Porter C.H., Boote K.J., Batchelor W.D., Hunt L.A., Wilkens P.W., Singh U., Gijsman A.J., Ritchie J.T. (2003). The DSSAT Cropping System Model. Eur. J. Agron..

[59] Government of Prince Edward Island Where Is Prince Edward Island?. https://www.princeedwardisland.ca/en/information/where-is-prince-edward-island.

[60] Government of Prince Edward Island PEI Population Report Quarterly. https://www.princeedwardisland.ca/en/information/finance/pei-population-report-quarterly.

[61] Government of Prince Edward Island Agriculture on PEI. https://www.princeedwardisland.ca/en/information/agriculture-and-land/agriculture-pei.

[62] United Potato Growers of Canada Canadian Potato Production. https://unitedpotatocanada.com/wp-content/uploads/2022/12/Canadian-Potato-Production-2022-12-07-2022.pdf.

[63] Jiang Y., Zebarth B., Love J. (2011). Long-Term Simulations of Nitrate Leaching from Potato Production Systems in Prince Edward Island, Canada. Nutr. Cycl. Agroecosyst..

[64] Government of Canada Historical Data-Climate-Environment and Climate Change Canada. https://climate.weather.gc.ca/historical_data/search_historic_data_e.html.

[65] NASA-POWER Prediction of Worldwide Energy. https://power.larc.nasa.gov/data-access-viewer/.

[66] PEI Potato Board Potatoes from Prince Edward Island | PEI Potatoes. https://www.peipotato.org/pei-potato-industry.

[67] Hausfather Z. CMIP6: The next Generation of Climate Models Explained. https://www.carbonbrief.org/cmip6-the-next-generation-of-climate-models-explained.

[68] Hausfather Z. Explainer: How ‘Shared Socioeconomic Pathways’ Explore Future Climate Change-Carbon Brief. https://www.carbonbrief.org/explainer-how-shared-socioeconomic-pathways-explore-future-climate-change/.

[69] O’Neill B.C., Tebaldi C., van Vuuren D.P., Eyring V., Friedlingstein P., Hurtt G., Knutti R., Kriegler E., Lamarque J.F., Lowe J. (2016). The Scenario Model Intercomparison Project (ScenarioMIP) for CMIP6. Geosci. Model Dev..

[70] Adavi Z., Moradi R., Saeidnejad A.H., Tadayon M.R., Mansouri H. (2018). Assessment of Potato Response to Climate Change and Adaptation Strategies. Sci. Hortic..

[71] Rahman A., Abdul Mojid M., Banu S. (2018). Climate Change Impact Assessment on Three Major Crops in the North-Central Region of Bangladesh Using DSSAT. Int. J. Agric. Biol. Eng..

[72] Sarkar R. (2009). Use of DSSAT to Model Cropping Systems. CAB Rev. Perspect. Agric. Vet. Sci. Nutr. Nat. Resour..

[73] Malkia R., Hartani T., Dechmi F. (2016). Evaluation of DSSAT Model for Sprinkler Irrigated Potato: A Case Study of Northeast Algeria. Afr. J. Agric. Res..

[74] Raymundo R., Asseng S., Prassad R., Kleinwechter U., Concha J., Condori B., Bowen W., Wolf J., Olesen J.E., Dong Q. (2017). Performance of the SUBSTOR-Potato Model across Contrasting Growing Conditions. Field Crops Res..

[75] Šťastná M., Toman F., Dufková J. (2010). Usage of SUBSTOR Model in Potato Yield Prediction. Agric. Water Manag..

[76] Šťastná M., Dufková J. (2008). Potato Simulation Model and Its Evaluation in Selected Central European Country. Agric. Conspec. Sci..

[77] Šťastná M., Oppeltová P., Dufková J. (2008). Validation of Potato Simulation Model. Acta Univ. Agric. Et Silvic. Mendel. Brun..

[78] Arora V.K., Nath J.C., Singh C.B. (2013). Analyzing Potato Response to Irrigation and Nitrogen Regimes in a Sub-Tropical Environment Using SUBSTOR-Potato Model. Agric. Water Manag..

[79] Goswami B., Hussain R., Kumar P.V., Saikia U.S., Banarjee S. (2018). Impact Assessment of Climate Change on Potato Productivity in Assam Using SUBSTOR-Potato Model. J. Agrometeorol..

[80] Kleinwechter U., Gastelo M., Ritchie J., Nelson G., Asseng S. (2016). Simulating Cultivar Variations in Potato Yields for Contrasting Environments. Agric. Syst..

[81] Prasad R., Hochmuth G.J., Boote K.J. (2015). Estimation of Nitrogen Pools in Irrigated Potato Production on Sandy Soil Using the Model SUBSTOR. PLoS ONE.

[82] Chicco D., Warrens M.J., Jurman G. (2021). The Coefficient of Determination R-Squared Is More Informative than SMAPE, MAE, MAPE, MSE and RMSE in Regression Analysis Evaluation. PeerJ Comput. Sci..

[83] Vashisht B.B., Nigon T., Mulla D.J., Rosen C., Xu H., Twine T., Jalota S.K. (2015). Adaptation of Water and Nitrogen Management to Future Climates for Sustaining Potato Yield in Minnesota: Field and Simulation Study. Agric. Water Manag..

[84] Zhong X., Dutta U. (2015). Engaging Nash-Sutcliffe Efficiency and Model Efficiency Factor Indicators in Selecting and Validating Effective Light Rail System Operation and Maintenance Cost Models. J. Traffic Transp. Eng..

[85] PEI Agricultural Insurance Corporation Agricultural Crops in Prince Edward Island. https://www.princeedwardisland.ca/en/information/executive-council-office/agricultural-insurance-corporation.

[86] Tooley B.E., Mallory E.B., Porter G.A., Hoogenboom G. (2021). Predicting the Response of a Potato-Grain Production System to Climate Change for a Humid Continental Climate Using DSSAT. Agric. For. Meteorol..

[87] Tang J., Xiao D., Bai H., Wang B., Liu D.L., Feng P., Zhang Y., Zhang J. (2020). Potential Benefits of Potato Yield at Two Sites of Agro-Pastoral Ecotone in North China Under Future Climate Change. Int. J Plant Prod..

[88] Xiao G., Zheng F., Qiu Z., Yao Y. (2013). Impact of Climate Change on Water Use Efficiency by Wheat, Potato and Corn in Semiarid Areas of China. Agric. Ecosyst. Environ..

[89] Tubiello F.N., Rosenzweig C., Goldberg R.A., Jagtap S., Jones J.W. (2002). Effects of Climate Change on US Crop Production: Simulation Results Using Two Different GCM Scenarios. Part I: Wheat, Potato, Maize, and Citrus. Clim. Res..

[90] Chang D.C., Sohn H.B., Cho J.H., Im J.S., Jin Y.I., Do G.R., Kim S.J., Cho H.M., Lee Y.B. (2014). Freezing and Frost Damage of Potato Plants: A Case Study on Growth Recovery, Yield Response, and Quality Changes. Potato Res..

[91] Chourasia K.N., Lal M.K., Tiwari R.K., Dev D., Kardile H.B., Patil V.U., Kumar A., Vanishree G., Kumar D., Bhardwaj V. (2021). Salinity Stress in Potato: Understanding Physiological, Biochemical and Molecular Responses. Life.

[92] Afzaal H., Farooque A.A., Abbas F., Acharya B., Esau T. (2020). Precision Irrigation Strategies for Sustainable Water Budgeting of Potato Crop in Prince Edward Island. Sustainability.

