# Supplementary material for: Assessing Future Climate Change Impacts on Potato Yields — A Case Study for Prince Edward Island, Canada
Source: Foods. 2023 Mar 10;12(6):1176. doi: 10.3390/foods12061176 (PMC10048153; doi:10.3390/foods12061176)
Supplement: Supplementary file 1 [file foods-12-01176-s001.zip › foods-2247372-supplementary.pdf]

Supplementary Information (S1)

Survey Form on PEI Potato Farming Practices

1. Indicate one potato variety (significant variety on your farm) you want to report about.

Check all that apply.

- ☐ Russet Burbank
- ☐ Prospect
- ☐ Dakota Russet
- ☐ Mountain Gem Russet
- ☐ Red Norland (or other table stock red variety)
- ☐ Eva (or other table stock white variety)
- ☐ Satina (or other table stock yellow variety)
- ☐ Creamer
- ☐ Other: ______________________

2. When do you usually start planting the chosen variety?

Check all that apply.

- ☐ April 1st – April 15th
- ☐ April 16th – April 30th
- ☐ May 1st – May 15th
- ☐ May 16th – May 31st
- ☐ June 1st – June 15th
- ☐ Other: ______________________

3. What was your farm's previous (last year) crop residue before planting thechosen variety?

Check all that apply.

- ☐ There were no previous plant residue
- ☐ Forage crops - incorporated green
- ☐ Forage crops - sprayed with glyphosate.
- ☐ Fall-seeded cover crops (grains, brassica)
- ☐ Corn stover
- ☐ Grain straw
- ☐ Other: ______________________

4. What is your target seed spacing for the chosen variety?

Check all that apply.

- [ ] < 8 inches
- [ ] 8 – 10 inches
- [ ] 10 – 12 inches
- [ ] 12 – 14 inches
- [ ] 14 – 16 inches
- [ ] > 16 inches

5. What is the planting depth (in inches) for the chosen variety?

Check all that apply.

- [ ] < 5
- [ ] 5 – 6
- [ ] 6 – 7
- [ ] 7 – 8
- [ ] 8 – 9
- [ ] > 9
- [ ] Other: ______________________________

6. What irrigation method do you use on your chosen variety?

Check all that apply.

- [ ] I do not irrigate on my farm
- [ ] Centre pivot.
- [ ] Reel and gun
- [ ] Travelling boom
- [ ] Drip.
- [ ] Other: ______________________________

7. How much water do you usually apply per irrigation application in mm on yourchosen variety?

Check all that apply.

- [ ] < 5.0 mm
- [ ] 5.1 – 10 mm
- [ ] 10.1 – 15 mm
- [ ] 15.1 – 20 mm

- [ ] 20.1 – 25 mm
- [ ] 25.1 – 30 mm
- [ ] > 30 mm
- [ ] Other: ____________________

8. How many times do you irrigate the chosen variety?

Check all that apply.

- [ ] 1 – 3 times
- [ ] 4 – 6 times
- [ ] 7 – 9 times
- [ ] 10 – 12 times
- [ ] > 12 times

9. What N fertilizer do you use on the chosen variety?

Check all that apply.

- [ ] Ammonium nitrate
- [ ] Calcium ammonium nitrate
- [ ] Calcium nitrate
- [ ] MESZ/DAP
- [ ] Urea ammonium nitrate
- [ ] ESN
- [ ] Super U/Agrotain
- [ ] Ammonium sulphate
- [ ] Foliar N products (Last N, SRN, e. t. c.)
- [ ] Urea
- [ ] Other: ____________________

10. What method was used to apply the nitrogen fertilizer to the chosen variety?

Check all that apply.

- [ ] Pre-plant broadcast
- [ ] Banded at planting
- [ ] Top dress at hilling.
- [ ] In irrigation water (fertigation)
- [ ] Foliar fertilizers
- [ ] Other: ____________________

11. What is the quantity of the nitrogen fertilizer that is applied from all sources to

the chosen variety?

Check all that apply.

☐ < 100 lbs/ac
☐ 100 – 150 lbs/ac
☐ 150 – 200 lbs/ac
☐ 200 – 250 lbs/ac
☐ Other: ____________________

12. How often was the nitrogen fertilizer applied to the chosen variety?

Check all that apply.

☐ 1 – 3 times
☐ 4 – 6 times
☐ 7 –10 times
☐ Other: ____________________

13. Did you apply manure or compost to the chosen variety?

Check all that apply.

☐ Compost or manure not applied
☐ Manure.
☐ Compost
☐ Other: ____________________

14. What is the quantity of manure/compost per application, per wet weight on the chosen variety?

Check all that apply.

☐ < 10 T/ac
☐ 10 – 20 T/ac
☐ > 20 T/ac
☐ Other: ____________________

15. How often do you apply the manure/compost on the chosen variety?

____

16. What is your primary form of tillage ahead of potato planting?

Check all that apply.

- ☐ Moldboard plough
- ☐ Discs
- ☐ Chisel plough
- ☐ Vertical (residue, lemkem, pottinger e. t. c.)
- ☐ Other: ______________________

17. How many times do you perform tillage ahead of potato planting?

Check all that apply.

- ☐ 1 time
- ☐ 2 times
- ☐ 3 times
- ☐ 4 times
- ☐ Other: ______________________

18. What is the tillage depth per application (inch)?

Check all that apply.

- ☐ < 6
- ☐ 6 – 8
- ☐ 8 – 10
- ☐ 10 –12
- ☐ > 12
- ☐ Other: ______________________

19. What is the typical harvest date for your chosen variety?

Check all that apply.

- ☐ Early September
- ☐ Mid September
- ☐ Late September
- ☐ Early October
- ☐ Mid October
- ☐ Late October
- ☐ Other: ______________________

20. How many days does it take for this variety to mature (Planting to harvest)?

Check all that apply.

- [ ] 90 – 100 days
- [ ] 101 – 120 days
- [ ] 121 – 130 days
- [ ] 131 – 140 days
- [ ] Other: ____________________

21. What is the average yield of the chosen potato variety in cwt/acre at harvest?

____

22. Kindly give the additional necessary information if there is any.

____

____
